# Supplementary material for: Mental Pain Correlates with Mind Wandering, Self-Reflection, and Insight in Individuals with Psychotic Disorders: A Cross-Sectional Study
Source: Brain Sci. 2023 Nov 7;13(11):1557. doi: 10.3390/brainsci13111557 (PMC10670292; doi:10.3390/brainsci13111557)
Supplement: Supplementary file 1 [file brainsci-13-01557-s001.zip › brainsci-2671500-supplementary.pdf]

Table S1. Age sensitivity.

Partial Correlation

		PAS-Total	MW-D	MW-S	MW-Total	SRIS-SR	SRIS-I	SRIS-Total
PAS-Total	Spearman's rho	—						
	p-value	—						
	N	—						
MW-D	Spearman's rho	0.451 *	—					
	p-value	0.031	—					
	N	31	—					
MW-S	Spearman's rho	0.620 **	0.748 ***	—				
	p-value	0.002	< .001	—				
	N	31	31	—				
MW-Total	Spearman's rho	0.627 **	0.949 ***	0.888 ***	—			
	p-value	0.001	< .001	< .001	—			
	N	31	31	31	—			
SRIS-SR	Spearman's rho	−0.491 *	−0.536 *	−0.577 **	−0.602 **	—		
	p-value	0.015	0.010	0.005	0.003	—		
	N	32	30	30	30	—		
SRIS-I	Spearman's rho	−0.738 ***	−0.370	−0.580 **	−0.513 *	0.567 **	—	
	p-value	< .001	0.082	0.004	0.012	0.004	—	
	N	33	31	31	31	32	—	
SRIS-Total	Spearman's rho	−0.442 *	−0.484 *	−0.540 **	−0.504 *	0.868 ***	0.644 ***	—
	p-value	0.031	0.023	0.009	0.017	< .001	< .001	—
	N	32	30	30	30	32	32	—

Note. Controlling for 'Gender', 'Years Of Illness', 'Education, Occupation, 'Marriage, 'Parental status', Housing arrangements', and 'Diagnosis'.

Note. * $p < 0.05$, ** $p < 0.01$, *** $p < 0.001$.

Table S2. Diagnosis Sensitivity.

Partial Correlation

		PAS-Total	MW-D	MW-S	MW-Total	SRIS-SR	SRIS-I	SRIS-Total
PAS-Total	Spearman's rho	—						
	p-value	—						
	N	—						
MW-D	Spearman's rho	0.676 ***	—					
	p-value	< .001	—					
	N	31	—					
MW-S	Spearman's rho	0.692 ***	0.785 ***	—				
	p-value	< .001	< .001	—				
	N	31	31	—				
MW-Total	Spearman's rho	0.811 ***	0.944 ***	0.914 ***	—			
	p-value	< .001	< .001	< .001	—			
	N	31	31	31	—			
SRIS-SR	Spearman's rho	−0.445 *	−0.473 *	−0.502 *	−0.527 *	—		
	p-value	0.029	0.026	0.017	0.012	—		
	N	32	30	30	30	—		
SRIS-I	Spearman's rho	−0.732 ***	−0.622 **	−0.670 ***	−0.720 ***	0.566 **	—	
	p-value	< .001	0.002	< .001	< .001	0.004	—	
	N	33	31	31	31	32	—	
SRIS-Total	Spearman's rho	−0.400	−0.548 **	−0.509 *	−0.525 *	0.888 ***	0.624 **	—
	p-value	0.053	0.008	0.016	0.012	< .001	0.001	—
	N	32	30	30	30	32	32	—

Note. Controlling for 'Gender', 'Years Of Illness', 'Education, Occupation, 'Marriage, 'Parental status', Housing arrangements', and 'Age'.

Note. * $p < 0.05$, ** $p < 0.01$, *** $p < 0.001$.

Table S3. Sensitivity housing arrangements.

Partial Correlation

		PAS-Total	MW-D	MW-S	MW-Total	SRIS-SR	SRIS-I	SRIS-Total
PAS-Total	Spearman's rho	—						
	p-value	—						
	N	—						
MW-D	Spearman's rho	0.598 **	—					
	p-value	0.003	—					
	N	31	—					
MW-S	Spearman's rho	0.605 **	0.808 ***	—				
	p-value	0.002	< .001	—				
	N	31	31	—				
MW-Total	Spearman's rho	0.694 ***	0.949 ***	0.923 ***	—			
	p-value	< .001	< .001	< .001	—			
	N	31	31	31	—			
SRIS-SR	Spearman's rho	−0.507 *	−0.528 *	−0.572 **	−0.581 **	—		
	p-value	0.012	0.011	0.005	0.005	—		
	N	32	30	30	30	—		
SRIS-I	Spearman's rho	−0.733 ***	−0.516 *	−0.550 **	−0.572 **	0.595 **	—	
	p-value	< .001	0.012	0.007	0.004	0.002	—	
	N	33	31	31	31	32	—	
SRIS-Total	Spearman's rho	−0.425 *	−0.492 *	−0.464 *	−0.449 *	0.856 ***	0.637 ***	—
	p-value	0.039	0.020	0.030	0.036	< .001	< .001	—
	N	32	30	30	30	32	32	—

Note. Controlling for 'Gender', 'Years Of Illness', 'Education, Occupation, 'Marriage, 'Parental status', 'Diagnosis', and 'Age'.

Note. * p < 0.05, ** p < 0.01, *** p < 0.001.

Table S4. Parental status sensitivity.

Partial Correlation

		PAS-Total	MW-D	MW-S	MW-Total	SRIS-SR	SRIS-I	SRIS-Total
PAS-Total	Spearman's rho	—						
	p-value	—						
	N	—						
MW-D	Spearman's rho	0.670 ***	—					
	p-value	< .001	—					
	N	31	—					
MW-S	Spearman's rho	0.675 ***	0.781 ***	—				
	p-value	< .001	< .001	—				
	N	31	31	—				
MW-Total	Spearman's rho	0.797 ***	0.942 ***	0.912 ***	—			
	p-value	< .001	< .001	< .001	—			
	N	31	31	31	—			
SRIS-SR	Spearman's rho	−0.503 *	−0.520 *	−0.554 **	−0.571 **	—		
	p-value	0.012	0.013	0.007	0.005	—		
	N	32	30	30	30	—		
SRIS-I	Spearman's rho	−0.734 ***	−0.601 **	−0.627 **	−0.677 ***	0.594 **	—	
	p-value	< .001	0.002	0.001	< .001	0.002	—	
	N	33	31	31	31	32	—	
SRIS-Total	Spearman's rho	−0.436 *	−0.577 **	−0.539 **	−0.550 **	0.880 ***	0.641 ***	—
	p-value	0.033	0.005	0.010	0.008	< .001	< .001	—
	N	32	30	30	30	32	32	—

Note. Controlling for 'Gender', 'Years Of Illness', 'Education, Occupation, 'Marriage, Housing arrangements', 'Diagnosis', and 'Age'.

Note. * $p < 0.05$, ** $p < 0.01$, *** $p < 0.001$.

Table S5. Marriage sensitivity.

Partial Correlation

		PAS-Total	MW-D	MW-S	MW-Total	SRIS-SR	SRIS-I	SRIS-Total
PAS-Total	Spearman's rho	—						
	p-value	—						
	N	—						
MW-D	Spearman's rho	0.627 **	—					
	p-value	0.001	—					
	N	31	—					
MW-S	Spearman's rho	0.663 ***	0.794 ***	—				
	p-value	< .001	< .001	—				
	N	31	31	—				
MW-Total	Spearman's rho	0.755 ***	0.950 ***	0.912 ***	—			
	p-value	< .001	< .001	< .001	—			
	N	31	31	31	—			
SRIS-SR	Spearman's rho	−0.531 **	−0.575 **	−0.593 **	−0.630 **	—		
	p-value	0.008	0.005	0.004	0.002	—		
	N	32	30	30	30	—		
SRIS-I	Spearman's rho	−0.736 ***	−0.541 **	−0.615 **	−0.631 **	0.599 **	—	
	p-value	< .001	0.008	0.002	0.001	0.002	—	
	N	33	31	31	31	32	—	
SRIS-Total	Spearman's rho	−0.442 *	−0.563 **	−0.550 **	−0.548 **	0.861 ***	0.645 ***	—
	p-value	0.031	0.006	0.008	0.008	< .001	< .001	—
	N	32	30	30	30	32	32	—

Note. Controlling for 'Gender', 'Years Of Illness', 'Education, Occupation, 'Parental status', Housing arrangements', 'Diagnosis', and 'Age'.

Note. * $p < 0.05$, ** $p < 0.01$, *** $p < 0.001$.

Table S6. Occupation sensitivity.

Partial Correlation

		PAS-Total	MW-D	MW-S	MW-Total	SRIS-SR	SRIS-I	SRIS-Total
PAS-Total	Spearman's rho	—						
	p-value	—						
	N	—						
MW-D	Spearman's rho	0.657 ***	—					
	p-value	< .001	—					
	N	31	—					
MW-S	Spearman's rho	0.677 ***	0.782 ***	—				
	p-value	< .001	< .001	—				
	N	31	31	—				
MW-Total	Spearman's rho	0.799 ***	0.943 ***	0.912 ***	—			
	p-value	< .001	< .001	< .001	—			
	N	31	31	31	—			
SRIS-SR	Spearman's rho	−0.491 *	−0.523 *	−0.559 **	−0.578 **	—		
	p-value	0.015	0.012	0.007	0.005	—		
	N	32	30	30	30	—		
SRIS-I	Spearman's rho	−0.728 ***	−0.616 **	−0.659 ***	−0.713 ***	0.595 **	—	
	p-value	< .001	0.002	< .001	< .001	0.002	—	
	N	33	31	31	31	32	—	
SRIS-Total	Spearman's rho	−0.414 *	−0.578 **	−0.538 **	−0.550 **	0.882 ***	0.632 ***	—
	p-value	0.044	0.005	0.010	0.008	< .001	< .001	—
	N	32	30	30	30	32	32	—

Note. Controlling for 'Gender', ‘Years Of Illness’, 'Education, 'Marriage, 'Parental status’, Housing arrangements’, ‘Diagnosis’, and 'Age'.

Note. * $p < 0.05$, ** $p < 0.01$, *** $p < 0.001$.

Table S7. Education sensitivity.

Partial Correlation

		PAS-Total	MW-D	MW-S	MW-Total	SRIS-SR	SRIS-I	SRIS-Total
PAS-Total	Spearman's rho	—						
	p-value	—						
	N	—						
MW-D	Spearman's rho	0.613 **	—					
	p-value	0.002	—					
	N	31	—					
MW-S	Spearman's rho	0.721 ***	0.745 ***	—				
	p-value	< .001	< .001	—				
	N	31	31	—				
MW-Total	Spearman's rho	0.802 ***	0.926 ***	0.911 ***	—			
	p-value	< .001	< .001	< .001	—			
	N	31	31	31	—			
SRIS-SR	Spearman's rho	−0.572 **	−0.491 *	−0.612 **	−0.605 **	—		
	p-value	0.003	0.020	0.002	0.003	—		
	N	32	30	30	30	—		
SRIS-I	Spearman's rho	−0.774 ***	−0.553 **	−0.700 ***	−0.713 ***	0.652 ***	—	
	p-value	< .001	0.006	< .001	< .001	< .001	—	
	N	33	31	31	31	32	—	
SRIS-Total	Spearman's rho	−0.500 *	−0.555 **	−0.589 **	−0.582 **	0.891 ***	0.681 ***	—
	p-value	0.013	0.007	0.004	0.004	< .001	< .001	—
	N	32	30	30	30	32	32	—

Note. Controlling for 'Gender', 'Years Of Illness', Occupation, 'Marriage, 'Parental status', Housing arrangements', 'Diagnosis', and 'Age'.

Note. * $p < 0.05$, ** $p < 0.01$, *** $p < 0.001$.

Table S8. Years of illness sensitivity

Partial Correlation

		PAS-Total	MW-S	MW-D	MW-Total	SRIS-SR	SRIS-I	SRIS-Total
PAS-Total	Spearman's rho	—						
	p-value	—						
	N	—						
MW-S	Spearman's rho	0.635 **	—					
	p-value	0.001	—					
	N	31	—					
MW-D	Spearman's rho	0.550 **	0.794 ***	—				
	p-value	0.007	< .001	—				
	N	31	31	—				
MW-Total	Spearman's rho	0.696 ***	0.911 ***	0.954 ***	—			
	p-value	< .001	< .001	< .001	—			
	N	31	31	31	—			
SRIS-SR	Spearman's rho	−0.505 *	−0.568 **	−0.494 *	−0.564 **	—	0.597 **	
	p-value	0.012	0.006	0.020	0.006	—	0.002	
	N	32	30	30	30	—	32	
SRIS-I	Spearman's rho	−0.730 ***	−0.573 **	−0.444 *	−0.557 **		—	
	p-value	< .001	0.004	0.034	0.006		—	
	N	33	31	31	31		—	
SRIS-Total	Spearman's rho	−0.405 *	−0.463 *	−0.388	−0.406	0.853 ***	0.627 **	—
	p-value	0.050	0.030	0.074	0.061	< .001	0.001	—
	N	32	30	30	30	32	32	—

Note. Controlling for 'Gender', 'Education, Occupation, 'Marriage, 'Parental status', Housing arrangements', 'Diagnosis', and 'Age'.

Note. * $p < 0.05$, ** $p < 0.01$, *** $p < 0.001$.

Table S9. Gender sensitivity.

Partial Correlation

		PAS-Total	MW-S	MW-D	MW-Total	SRIS-I	SRIS-SR	SRIS-Total
PAS-Total	Spearman's rho	—						
	p-value	—						
	N	—						
MW-S	Spearman's rho	0.685 ***	—					
	p-value	< .001	—					
	N	31	—					
MW-D	Spearman's rho	0.669 ***	0.781 ***	—				
	p-value	< .001	< .001	—				
	N	31	31	—				
MW-Total	Spearman's rho	0.805 ***	0.911 ***	0.944 ***	—			
	p-value	< .001	< .001	< .001	—			
	N	31	31	31	—			
SRIS-I	Spearman's rho	−0.726 ***	−0.647 ***	−0.587 **	−0.684 ***	—		
	p-value	< .001	< .001	0.003	< .001	—		
	N	33	31	31	31	—		
SRIS-SR	Spearman's rho	−0.506 *	−0.569 **	−0.519 *	−0.584 **	0.584 **	—	
	p-value	0.012	0.006	0.013	0.004	0.003	—	
	N	32	30	30	30	32	—	
SRIS-Total	Spearman's rho	−0.431 *	−0.546 **	−0.577 **	−0.559 **	0.605 **	0.877 ***	—
	p-value	0.036	0.009	0.005	0.007	0.002	< .001	—
	N	32	30	30	30	32	32	—

Note. Controlling for 'Years Of Illness', 'Education, Occupation, 'Marriage, 'Parental status', Housing arrangements', 'Diagnosis', and 'Age'.

Note. * $p < 0.05$, ** $p < 0.01$, *** $p < 0.001$.

Table S10. Correlation Matrix of potential confounders only.

Correlation Matrix

		Gender	Year of Illness	Education	Occupation	Marriage	Parental status	Housing Arrangements	Diagnosis	Age
Gender	Spearman's rho	—								
	df	—								
	p-value	—								
Year of Illness	Spearman's rho	0.170	—							
	df	32	—							
	p-value	0.336	—							
Education	Spearman's rho	0.022	−0.052	—						
	df	32	32	—						
	p-value	0.901	0.769	—						
Occupation	Spearman's rho	−0.296	−0.230	0.135	—					
	df	32	32	32	—					
	p-value	0.089	0.190	0.447	—					
Marriage	Spearman's rho	0.061	0.127	−0.009	0.161	—				
	df	32	32	32	32	—				
	p-value	0.732	0.473	0.959	0.362	—				
Parental status	Spearman's rho	0.219	0.014	−0.193	0.228	0.364 *	—			
	df	32	32	32	32	32	—			
	p-value	0.213	0.937	0.274	0.195	0.034	—			
Housing Arrangements	Spearman's rho	−0.058	0.027	0.256	0.018	−0.692 ***	−0.609 ***	—		
	df	32	32	32	32	32	32	—		
	p-value	0.746	0.878	0.144	0.917	< .001	< .001	—		
'Diagnosis'	Spearman's rho	−0.219	0.075	−0.274	−0.013	−0.106	−0.433 *	0.195	—	
	df	32	32	32	32	32	32	32	—	
	p-value	0.213	0.675	0.117	0.943	0.550	0.010	0.270	—	
Age	Spearman's rho	0.190	0.450 **	−0.322	−0.297	0.000	0.251	−0.255	−0.186	—
	df	32	32	32	32	32	32	32	32	—
	p-value	0.281	0.008	0.063	0.088	1.000	0.151	0.145	0.291	—

Note. * $p < 0.05$, ** $p < 0.01$, *** $p < 0.001$.

Table S11. Correlation Matrix of potential confounders and scale's measures.

		PAS-Total	MW-D	MW-S	MW-Total	SRIS-Total	SRIS-I	SRIS-SR	Age	Gender	Years Of Illness	Education	Occupation	Marriage	Parental status	Housing Arrangements
PAS-Total	Spearman's rho	—														
	df	—														
	p-value	—														
MW-D	Spearman's rho	0.409 *	—													
	df	29	—													
	p-value	0.022	—													
MW-S	Spearman's rho	0.577 ***	0.734 ***	—												
	df	29	29	—												
	p-value	< .001	< .001	—												
MW-Total	Spearman's rho	0.567 ***	0.937 ***	0.895 ***	—											
	df	29	29	29	—											
	p-value	< .001	< .001	< .001	—											
SRIS-Total	Spearman's rho	−0.476 **	−0.276	−0.328	−0.282	—										
	df	30	28	28	28	—										
	p-value	0.006	0.139	0.077	0.132	—										
SRIS-I	Spearman's rho	−0.753 ***	−0.235	−0.483 **	−0.363 *	0.624 ***	—									
	df	31	29	29	29	30	—									
	p-value	< .001	0.204	0.006	0.044	< .001	—									
SRIS-SR	Spearman's rho	−0.553 **	−0.309	−0.400 *	−0.372 *	0.896 ***	0.632 ***	—								
	df	30	28	28	28	30	30	—								
	p-value	0.001	0.097	0.029	0.043	< .001	< .001	—								
Age	Spearman's rho	−0.123	0.280	−0.013	0.198	−0.048	0.210	−0.022	—							
	df	32	29	29	29	30	31	30	—							
	p-value	0.488	0.127	0.944	0.285	0.794	0.241	0.906	—							

		PAS-Total	MW-D	MW-S	MW-Total	SRIS-Total	SRIS-I	SRIS-SR	Age	Gender	Years Of Illness	Education	Occupation	Marriage	Parental status	Housing Arrangements
Gender	Spearman's rho	−0.041	0.103	−0.025	0.095	0.047	0.368 *	0.110	0.190	—						
	df	32	29	29	29	30	31	30	32	—						
	p-value	0.818	0.580	0.895	0.612	0.798	0.035	0.550	0.281	—						
Year of Illness	Spearman's rho	−0.127	−0.214	−0.212	−0.195	−0.182	0.127	−0.066	0.450 **	0.170	—					
	df	32	29	29	29	30	31	30	32	32	—					
	p-value	0.473	0.248	0.253	0.294	0.318	0.481	0.719	0.008	0.336	—					
Education	Spearman's rho	−0.327	−0.073	−0.210	−0.172	0.396 *	0.348 *	0.441 *	−0.322	0.022	−0.052	—				
	df	32	29	29	29	30	31	30	32	32	32	—				
	p-value	0.059	0.696	0.256	0.354	0.025	0.047	0.011	0.063	0.901	0.769	—				
Occupation	Spearman's rho	0.028	0.039	0.104	0.043	−0.164	−0.160	−0.164	−0.297	−0.296	−0.230	0.135	—			
	df	32	29	29	29	30	31	30	32	32	32	32	—			
	p-value	0.873	0.835	0.578	0.818	0.368	0.374	0.369	0.088	0.089	0.190	0.447	—			
Marriage	Spearman's rho	0.213	0.011	−0.135	−0.059	−0.308	−0.124	−0.299	0.000	0.061	0.127	−0.009	0.161	—		
	df	32	29	29	29	30	31	30	32	32	32	32	32	—		
	p-value	0.227	0.954	0.469	0.752	0.086	0.490	0.097	1.000	0.732	0.473	0.959	0.362	—		
Parental status'	Spearman's rho	0.070	0.214	0.066	0.184	−0.169	0.059	−0.061	0.251	0.219	0.014	−0.193	0.228	0.364 *	—	
	df	32	29	29	29	30	31	30	32	32	32	32	32	32	—	
	p-value	0.693	0.248	0.723	0.323	0.355	0.746	0.738	0.151	0.213	0.937	0.274	0.195	0.034	—	
Housing Arrangements	Spearman's rho	−0.195	−0.069	0.182	0.060	0.298	0.100	0.204	−0.255	−0.058	0.027	0.256	0.018	−0.692 ***	−0.609 ***	—
	df	32	29	29	29	30	31	30	32	32	32	32	32	32	32	—
	p-value	0.270	0.711	0.328	0.747	0.098	0.579	0.264	0.145	0.746	0.878	0.144	0.917	< .001	< .001	—
Diagnosis	Spearman's rho	0.080	−0.259	−0.135	−0.231	−0.343	−0.259	−0.436 *	−0.186	−0.219	0.075	−0.274	−0.013	−0.106	−0.433 *	0.195
	df	32	29	29	29	30	31	30	32	32	32	32	32	32	32	32
	p-value	0.655	0.159	0.469	0.210	0.054	0.146	0.013	0.291	0.213	0.675	0.117	0.943	0.550	0.010	0.270

References

[1] The jamovi project (2022). *jamovi*. (Version 2.3) [Computer Software]. Retrieved from https://www.jamovi.org.

[2] R Core Team (2021). *R. A Language and environment for statistical computing*. (Version 4.1) [Computer software]. Retrieved from https://cran.r-project.org. (R packages retrieved from MRAN snapshot 2022−01−01).

[3] Revelle, W. (2019). *psych. Procedures for Psychological, Psychometric, and Personality Research*. [R package]. Retrieved from https://cran.r-project.org/package=psych.

[4] Kim, S. (2015). *ppcor. Partial and Semi-Partial (Part) Correlation*. [R package]. Retrieved from https://cran.r-project.org/package=ppcor.
